# Are They Created Equal? A Relative Weights Analysis of the Contributions of Job Demands and Resources to Well-Being and Turnover Intention

**DOI:** 10.1177/00332941221103536

**Published:** 2022-06-16

**Authors:** Corey Hoare, Christian Vandenberghe

**Affiliations:** 10014 HEC Montréal, Montréal, QC, Canada

**Keywords:** job demands-resources model, personal resources, emotional exhaustion, turnover intention, relative weights analysis

## Abstract

Building upon the Job Demands-Resources (JD-R) model (Demerouti et al., 2001) and the extensive research on employee turnover intention and well-being, we examined various demands and resources in relation to these outcomes. This study examined the differential relationship between job demands, and personal and job resources, and two organizational outcomes: turnover intention and emotional exhaustion. The job demands were role overload, role conflict, role ambiguity, and work-life balance. The job resources were resilience, servant leadership, relatedness, autonomy, job opportunities, pay satisfaction, and person-organization fit. An online questionnaire was administered to full-time employees via Qualtrics panel (*N* = 364). Job demands were positively related to emotional exhaustion, and personal and job resources were negatively related to turnover intention. Using relative weights analysis, demands and resources were found to account for different amounts of variance in the outcome variables. This study informs our understanding of and contributes to the advancement of the JD-R model to encompass various job demands and personal and job resources and their differential relationship to emotional exhaustion and turnover intention.

A recent survey by consulting firm Deloitte noted that over three quarters (77%) of full-time professionals in the U.S. reported feelings of burnout linked to their current job (Deloitte, 2018). The same survey notes that nearly a third of employees surveyed reported that lack of support from superiors, time pressure and long work hours contribute to their feelings of burnout (31%, 30%, and 29%, respectively). The survey also reports that nearly 4.2 in 10 employees have voluntarily left a job because they felt high levels of burnout. Understandably, the factors mentioned above have been found to be strongly correlated with burnout (Lee & Ashforth, 1996) as well as with turnover (Griffeth et al., 2000). Though the relationship may be more nuanced as various job or work-related characteristics have been found to interact, and in certain instances, alleviate or prevent burnout (Bakker et al., 2005).

Given that various factors have been found to influence employees’ burnout and voluntary turnover, the purpose of the present paper is to investigate the relationship between various job, organizational and personal characteristics and burnout and turnover intention. The present study, as described in the Job Demands-Resources (JD-R) model (Demerouti et al., 2001), is based on the idea that negative job-related characteristics (i.e., job demands) should contribute to emotional exhaustion while positive job-related characteristics (i.e., job resources) should reduce disengagement. Our purpose is to examine the relative importance of demands and resources to predict emotional exhaustion and turnover intention as specific work outcomes, which previous research has not addressed. A recent review by Schaufeli and Taris (2014) noted that most studies that used the JD-R model (Demerouti et al., 2001) assumed that individual demands and resources are created equal. The purpose of this article is twofold: (1) test the revised JD-R model with emotional exhaustion and turnover intention as the outcomes and (2) determine the relative importance of each demand and resource to predict the target outcomes. In isolating the individual contributions of each demand and resource, the present study contributes to our understanding of the JD-R model (Bakker & Demerouti, 2014, 2017; Demerouti et al., 2001).

## Theoretical Background

### The Job Demands-Resources Model

The JD-R model (Demerouti et al., 2001) has been depicted as a model of employee well-being (Schaufeli & Taris, 2014), wherein the basic postulates are that (1) job demands are commonly related to health ailments (i.e., exhaustion) and (2) job resources are commonly related to motivational outcomes (i.e., engagement; Bakker & Demerouti, 2014). Initially, the JD-R model (i.e., Demerouti et al., 2001) did not account for the antecedents or processes underlying job demands and resources and its outcomes (e.g., exhaustion and disengagement; Schaufeli & Taris, 2014). However, the model has been widely validated using longitudinal (e.g., Schaufeli et al., 2009) and cross-lagged (e.g., Hakanen et al., 2008) designs, and among cross-national (e.g., Brough et al., 2013) and international (e.g., Xanthopoulou et al., 2007) samples. The extensive research on the model has led some to suggest that the JD-R model can be envisioned as a theory (e.g., Bakker & Demerouti, 2014). When various job and workplace characteristics are analyzed using the model, one can elucidate their impact on employees (e.g., well-being, motivation, and job performance) and make predictions based on the perceived level of demands and resources (Bakker & Demerouti, 2014).

The demands to exhaustion pathway, referring to the health impairment process (or strain process: Brough et al., 2013), is due to job demands draining individuals’ ability to cope with them (Bakker & Demerouti, 2014; Bakker et al., 2003). The resources to disengagement pathway, referring to the motivational process, occurs due to job resources being important for the satisfaction of an individual’s basic psychological needs (Bakker et al., 2003; Deci & Ryan, 2000; Ryan & Deci, 2000). Outside the JD-R model (Demerouti et al., 2001), job demands and resources have been found to be related to various outcomes. Job demands were found to be positively related to work-home interference (DiRenzo et al., 2011) and work-family conflict (Hall et al., 2010) and negatively related to the satisfaction of basic needs (Van den Broeck et al., 2008). Job resources have been found to be negatively related to work-home interference (Bakker et al., 2011) and positively related to the satisfaction of basic needs (Van den Broeck et al., 2008) and work engagement (Hakanen et al., 2008; Xanthopoulou et al., 2007).

Following a prolonged period where job resources are lacking, employees will feel the draining effects of job demands (e.g., Bakker et al., 2003). Consequently, employees may exhibit job withdrawal behaviors to shelter themselves against exhaustion (Bakker et al., 2005; Demerouti et al., 2001). At play are also the possible interactions between job demands and job resources. An abundance of resources equips employees with the necessary tools to handle the impact of job demands. Specifically, employees reporting high levels of job demands and low levels of job resources reported elevated feelings of being exhausted (Bakker et al., 2005). Similarly, Hu and colleagues (2011) found that employees reporting elevated levels of job demands (e.g., workload) also reported feelings of burnout which led to a greater intention to leave the company. Elevated levels of job resources (e.g., job control) were associated with higher engagement and lower intention to leave. In several instances, job resources were found to be negatively related to exhaustion (Hakanen et al., 2008; Hu et al., 2011; Van den Broeck et al., 2010). Job resources and personal resources have been found to be positively related, and the latter was found to be negatively related to exhaustion (Xanthopoulou et al., 2007). Recently, Bakker and Demerouti (2017) proposed that personal and job resources may be complementary.

Upon reviewing the empirical and theoretical literature related to the two outcome variables, we observe that from a theoretical perspective they are distinct phenomena. Specifically, emotional exhaustion is defined as a point where “as emotional resources are depleted, workers feel they are no longer able to give of themselves at a psychological level” (Maslach et al., 1996, p. 192). In contrast, turnover intention is defined as the “intention of quitting, which would lead to turnover” (Michaels & Spector, 1982, p. 54). Thus, emotional exhaustion is essentially an emotional experience of the lack of inner resources for facing job demands while turnover intention refers to a cognition-based withdrawal tendency. Furthermore, to the best of our knowledge, no empirical work using the JD-R model has combined the two outcomes into an aggregate. Therefore, to remain consistent with extant literature and theory, we opted to maintain the two variables as distinct in our models.

As one can ascertain, the JD-R model is flexible in its definition of demands and resources, as will be described below, and to which outcome either is related (Bakker & Demerouti, 2017; Schaufeli & Taris, 2014). The flexibility, such that a wide range of job, workplace, organizational and personal characteristics may be related to an employee’s well-being and engagement, contributes to the theoretical value of the model (Schaufeli & Taris, 2014). Unsurprisingly, the composition of the JD-R model varies across organizational settings, types of employees, and industries and professions (Schaufeli & Taris, 2014).

### Job Demands

Job demands “refer to those physical, social, or organizational aspects of the job that require sustained physical or mental effort” (Demerouti et al., 2001, p. 501), particularly role stressors and issues related to work-life balance.

#### Role Stressors

Employees face various stressors throughout the workday, commonly known as the three role stressors: role overload, role conflict and role ambiguity (King & King, 1990; Rizzo et al., 1970; Schaubroeck et al., 1989). In line with the JD-R model (Demerouti et al., 2001), role stressors can be conceptualized as demands given that they often elicit a negative appraisal from employees (Schaufeli & Taris, 2014). Role stressors differ from other job demands, such as emotional demands (e.g., Albrecht, 2015), cognitive demands (e.g., Bakker et al., 2011), and organizational changes (Xanthopoulou et al., 2007) as they are relatively linked to one another and focus on the individual’s work role (e.g., Bowling et al., 2017).

Role overload reflects the perception that one has too many responsibilities given the available resources (Schaubroeck et al., 1989). Role conflict occurs when the employee is exposed to conflicting expectations from superiors or organizational actors (Bowling et al., 2017). Role ambiguity refers to situations where tasks are ambiguously defined, and instructions are unclear (King & King, 1990; Rizzo et al., 1970). The three role stressors have been found to be positively related to one another (Bowling et al., 2017; Eatough et al., 2011), and to positively relate to turnover intention (Fried et al., 2008) and emotional exhaustion (Örtqvist & Wincent, 2006). They have also been found to be negatively related to job satisfaction (Bowling et al., 2017) and organizational commitment (Örtqvist & Wincent, 2006). Thus, we propose the following constructive replication hypothesis.

Hypotheses 1, 2 and 3 Role overload (H1), role ambiguity (H2), and role conflict (H3) will be positively related to (a) turnover intention and (b) emotional exhaustion.

#### Work-Life Balance

Work-family conflict indicates the degree of perceived conflict between various roles an employee occupies (e.g., work role, family role; Greenhaus & Beutell, 1985). An imbalance between roles may reduce resources and cause a stress reaction (e.g., exhaustion or turnover intention; Grandey & Cropanzano, 1999). Given relatively restrictive definitions of work-family conflict, Keeney and colleagues (2013) furthered Greenhaus and Beutell’s (1985) definition by presenting the concept of work interfering with life, which describes how an individual’s work role may reduce their involvement in other roles. Similarly, Brough and colleagues (2014) defined work-life balance as “an individual’s subjective appraisal of the accord between his/her work and non-work activities and life more generally” (p. 2728).

Work interfering with life and work-family conflict have been found to be positively related to exhaustion (Reichl et al., 2014; Zhang et al., 2012) and turnover intention (Boyar et al., 2003; Keeney et al., 2013). Work-life balance was found to be negatively related to turnover intention (Brough et al., 2014) and positively related to job satisfaction (Keeney et al., 2013), and family-work conflict has been found to be positively related to turnover intention (Zhang et al., 2012).

Hypothesis 4 Work-life balance (i.e., indicative of a good balance) will be negatively related to (a) turnover intention and (b) emotional exhaustion.

Given that job demands have been found to be more commonly (positively) related to exhaustion (e.g., Bakker & Demerouti, 2014), one would expect them to be of greater weight (than resources) in terms of relative importance.

Hypothesis 5 Job demands will account for the most variance in emotional exhaustion.

### Resources

Job resources “refer to those physical, psychological, social, or organizational aspects of the job that may do any of the following: (a) be functional in achieving work goals; (b) reduce job demands and the associated physiological or psychological costs; (c) stimulate personal growth and development” (Demerouti et al., 2001, p. 501). This definition suggests that employees value resources, which is a modification that has been proposed by Schaufeli and Taris (2014). Simply put, resources permit an employee to be psychologically engaged in their tasks (Bakker & Demerouti, 2007). Many resources have been examined such as resilience (Upadyaya et al., 2016), leadership (Nahrgang et al., 2011), social climate (Schaufeli & Taris, 2014), job autonomy (Bakker et al., 2005; DiRenzo et al., 2011; Van den Broeck et al., 2010), opportunities for professional development (Bakker et al., 2004), pay satisfaction (Crawford et al., 2010; Schaufeli & Taris, 2014), and person-organization fit (Jin et al., 2018; Mackey et al., 2017).

Several researchers noted the importance of differentiating personal from job resources. Personal resources are individual characteristics that help employees interact with the environment and relate to their resiliency while job resources refer to aspects of the work environment that alleviate the negative impact of job demands, facilitate work tasks, and aid in professional development (Demerouti & Bakker, 2011). Personal and job resources impact the workplace and influence employees’ learning, development, and well-being (Schaufeli & Taris, 2014). Job resources comprise structural job resources and social resources (Tims et al., 2012). The former refers to resources such as opportunities for development and autonomy, which permit employees to increase their knowledge, grow within the position and apply their knowledge. The latter refers to resources such as social support and supervisory coaching, which bolster employee relatedness (Tims et al., 2012). As such, job resources influence emotional exhaustion, such that when job resources increase, emotional exhaustion decreases (e.g., Bakker et al., 2004; Van den Broeck et al., 2010).

#### Conservation of Resources Theory

Similar to the JD-R model (Demerouti et al., 2001), Conversation of Resources (COR) theory (Hobfoll, 1989) proposes that individuals “strive to retain, protect, and build resources” (p. 513) with the loss or reduction of these resources being a threat to their well-being (Hobfoll, 1989; Hobfoll et al., 2003). COR theory identifies four types of resources: objects (e.g., a home), conditions (e.g., tenure), personal characteristics (e.g., resilience) and energies (e.g., knowledge; Hobfoll, 1989). Resource loss in the workplace may lead employees to want to quit their position to regain the lost resources or prevent further resource loss (Grandey & Cropanzano, 1999; Hobfoll, 1989). COR theory and the JD-R model face a similar critique in part given the broad definition of what a resource is (Halbesleben et al., 2014). This potential issue is further nuanced given that generally, a resource is inherently positive (i.e., beneficial). As such, anything beneficial in the eyes of an employee could be viewed as a resource (Gorgievski et al., 2011; Halbesleben & Wheeler, 2015). Along this line, Halbesleben et al. (2014) considered resources to be “anything perceived by the individual to help attain his or her goals” (p. 1338).

#### Personal Resource: Resilience

Stress may arise from various contexts and over prolonged periods (e.g., Ganster & Rosen, 2013). Individuals possess varying abilities relating to how they recuperate from stressful periods or a stressful event, which is known as resilience (Smith et al., 2008). The definition proposed by Smith and colleagues (2008) encompasses previous conceptualizations of resilience, where resilience is a process of overcoming stress and adverse events through the use of resilient qualities (e.g., support systems and self-esteem; Richardson, 2002). As resilience relates to the ability to recuperate from stressful periods, unsurprisingly it has been found to predict reduced burnout (e.g., emotional exhaustion; Upadayaya et al., 2016).

Hypothesis 6 Resilience will be negatively related to (a) turnover intention and (b) emotional exhaustion.

#### Job Resources: Basic Psychological Needs

Humans are motivated in a variety of ways. Self-determination theory (SDT; Ryan & Deci, 2000; Ryan et al., 1997) posits that in the organizational context, an employee’s work performance and personal well-being are influenced by the motivation (autonomous vs. controlled) oriented towards various aspects of their job. The satisfaction of the three basic needs of autonomy, competence, and relatedness has been found to mediate the relationship between an individual’s job characteristics and personal well-being (e.g., Deci et al., 2017). The three basic needs and their relevance to psychological growth and physical and mental well-being have been investigated (Ryan & Deci, 2000; Van den Broeck et al., 2016). As such, they appear to fit with the definition of resources in the JD-R model (e.g., Demerouti et al., 2001; Schaufeli & Taris, 2014).

In a review of SDT and work motivation, Gagné and Deci (2005) suggested that when a workplace facilitates the satisfaction of the three basic needs, employees will report higher job satisfaction and well-being and reduced role stress. The facilitation of need satisfaction strengthens employees’ motivation to complete their tasks, which leads to positive aftereffects (Gagné & Deci, 2005). The opposite occurs when need satisfaction is obstructed, such that less satisfaction of the basic needs relates to more negative aftereffects. One of these aftereffects could be turnover intention. It has been noted that while autonomy and relatedness were negatively related to turnover intention, competence was positively related to it (Van den Broeck et al., 2016). This diverging result may occur since a competent employee could easily transition to a different organization (Fugate et al., 2004). All three needs were noted as being positively related to well-being (Brien et al., 2012). Similarly, the basic needs negatively relate to emotional exhaustion (Albrecht, 2015; Van den Broeck et al., 2008).

Hypotheses 7 and 8 Relatedness (H7) and autonomy (H8) will be negatively related to (a) turnover intention and (b) emotional exhaustion.

#### Job Resources: Servant Leadership

In their review, Avolio et al. (2009) discuss several leadership models such as authentic, transformational, ethical, or servant leadership, and leader-member exchange (LMX). Leaders impact subordinates through their leadership style or behaviors (e.g., Reina et al., 2018). For example, Harms et al. (2017) found that transformational leadership and LMX were negatively related to subordinate stress and burnout, while abusive leadership was positively related to these outcomes. In the context of the JD-R model (Demerouti et al., 2001), servant leadership may be unique as it “stresses personal integrity and focuses on forming strong long-term relationships with employees” (Liden et al., 2008, p. 162). Servant leaders achieve this relationship through communication with employees to thoroughly understand their needs (Liden et al., 2008). Viewed as a resource, the presence of a servant leader could impact subordinate turnover intention and well-being (i.e., emotional exhaustion). Corroborating this view, when servant leadership was applied at the organizational level (e.g., retail stores), this idea of a serving culture was found to be negatively related to turnover intention (Liden et al., 2014). Additionally, servant leadership was found to be negatively related to burnout (Upadyaya et al., 2016).

Hypothesis 9 Servant leadership will be negatively related to (a) turnover intention and (b) emotional exhaustion.

#### Job Resources: Job Opportunities

Job attributes refer to a multitude of categories such as opportunities (e.g., promotion, learning, professional development, and growth), financial rewards (e.g., earnings and benefits) and locational advantages (e.g., leisure activities, social life; Konrad et al., 2000; Turban et al., 1992). Job attributes conceptualized as opportunities have been found to be positively related to an employee’s relocation decision acceptance (Turban et al., 1992). Similarly, opportunities for professional development paired with influence at work as job resources have been found to be negatively related to turnover intention (Van der Heijden et al., 2018). Opportunities to learn has been categorized as a job resource in several studies (e.g., Schaufeli et al., 2009; Van der Heijden et al., 2018). As part of a job resources category, opportunities to learn and develop were found to be negatively related to burnout (Schaufeli et al., 2009). As such, they will likely be more strongly related to turnover intention (e.g., the motivational process) rather than emotional exhaustion (e.g., the health impairment process).

Hypothesis 10 Job opportunities will be negatively related to (a) turnover intention and (b) emotional exhaustion.

#### Job Resources: Pay Satisfaction

Heneman and Schwab (1985) identified four dimensions of pay satisfaction: level, benefits, raise and structure/administration. The first, pay level satisfaction, as they describe, refers to an employee’s satisfaction with their current salary, its size, their take-home pay, and overall level of pay. As one would expect, actual pay (e.g., pay level) has been found to be positively related to overall satisfaction with pay (Judge et al., 2010). Though, an employee’s perception of their pay, rather than the actual dollar amount, relates more strongly to an employee’s behavior (Williams et al., 2006). One could view pay satisfaction as being the positive response to the level of financial rewards. According to Schaufeli and Taris (2014), pay level satisfaction can be conceptualized as a job resource. Perceptions and behaviors resulting from pay satisfaction may include turnover intention and actual turnover, with pay satisfaction found to be negatively related to both (Currall et al., 2005; Williams et al., 2006).

Hypothesis 11 Pay satisfaction will be negatively related to (a) turnover intention and (b) emotional exhaustion.

#### Job Resources: Person-Organization Fit

The congruence between an employee’s values and those of the company is known as person-organization fit (Chatman, 1989). Person-organization fit relates to an individual’s perception of their organization’s values. Person-organization fit can be viewed as a resource (e.g., Mackey et al., 2017) as it may provide employees reprieve from the stress of job demands (e.g., role overload; Edwards, 2008). An employee who perceives a strong fit with the organization and faces significant job demands may better be able to handle the latter’s draining effects as their values are reflected in the organization. Person-organization fit has been found to be negatively related to turnover intention (Jin et al., 2018) and emotional exhaustion (Siegall & McDonald, 2004).

Hypothesis 12 Person-organization fit will be negatively related to (a) turnover intention and (b) emotional exhaustion.

### Relative Importance of the Predictors

Regression analysis commonly used in organizational science does not partition the variance explained in the dependent variable by each independent variable. Relative-importance analysis (LeBreton et al., 2007) partitions the variance explained in the dependent variable by each predictor variable, even when predictors are correlated. Briefly, the relative importance of a variable encompasses said variable’s unique portion of the variance accounted for in the regression model (R^2^) while also considering the unique portion accounted for by other predictors (Johnson, 2000; LeBreton et al., 2007). As such, relative importance analysis is complementary to regression analysis (Tonidandel & LeBreton, 2011). In the context of the present study, relative importance analysis has several advantages. First, in the scope of the JD-R model (Demerouti et al., 2001), there are many possible demands and resources, which are likely correlated with one another. Second, with regression analysis alone, it would be challenging to attempt to differentiate several predictors’ individual contributions and whether they are statistically significant (Tonidandel et al., 2009). Relative importance analysis using the tool (i.e., RWA Web) created by Tonidandel and LeBreton (2015) addresses these concerns (e.g., Tonidandel & LeBreton, 2011).

First, in using RWA Web (Tonidandel & LeBreton, 2015), users are provided with the R^2^ for the model, the raw relative weight, rescaled relative weight, confidence intervals around the raw weights and confidence intervals test of significance. The most important distinction being between raw relative weights and rescaled relative weights. The former refers to “an additive decomposition of the total model R^2^ and can be interpreted as the proportion of variance in [the criterion] that is appropriately attributed to each [predictor]” (LeBreton et al., 2007; Tonidandel & LeBreton, 2015, p. 123). The latter refers to the value calculated by taking the raw relative weight and dividing it by the variance accounted for by the model to yield a proportion attributable to the predictor in the variance explained in the criterion (Tonidandel & LeBreton, 2015). In sum, by supplementing multiple regression analysis with relative importance analysis, the present study would be among the first to distinguish the relative importance of various demands and resources on work outcomes (i.e., turnover intention and emotional exhaustion).

## Methods

### Sample and Procedure

This study utilized an online questionnaire administered through Qualtrics (Qualtrics, 2019) to panel participants across the United States. Randomly selected members of the Qualtrics online panel received an email with a brief description of the study and eligibility requirements for participation. These requirements included that they (1) are employed on a full-time basis, (2) do not work exclusively from home, (3) are not self-employed and (4) have a few coworkers. There were no constraints relating to industry, company size or position. Before beginning the questionnaire, participants were met with a consent form outlining the context of participation and were given the choice to participate (or not). After completing the survey, participants were compensated the equivalent of US $2.50 in points that could be used towards the purchase of gift cards. All data were anonymous. A total of 377 participants completed the survey, though 13 responses were discarded from the primary analysis given significant missing data (>25%). The retained sample (*n* = 364) was primarily (78%) female. The average age was 41.53 years (SD = 12.41), and the average organizational tenure was 8.95 years (SD = 6.12). Participants worked on average 42.08 hours per week (SD = 5.41) with an average of 5.30 hours of overtime per week (SD = 7.20). Nearly three-fourths of the sample (*n* = 277) had at least a high school diploma.

### Measures

A five-point scale was used for all items. Unless otherwise specified, the scale anchor points were strongly disagree (1) to strongly agree (5).

### Work Outcomes

#### Turnover Intention

Proposed by Michaels and Spector (1982) and elaborated by Cohen (1999), the three-item measure evaluates participants’ intention to quit their organization. An example item is “I think a lot about leaving this organization” (α = .94).

#### Emotional Exhaustion

Emotional exhaustion was assessed using the five highest loading items of the emotional exhaustion scale of the original Maslach Burnout Inventory: General Survey (MBI-GS; Schaufeli et al., 1996) that were validated by Lapointe et al. (2011). A five-point Likert type scale ranging from never (1) to everyday (5) was used. An example item is “These days, I feel used up at the end of a work-day.” The original and reduced emotional exhaustion scales from the MBI-GS (Schaufeli et al., 1996) demonstrated high internal consistency (e.g., α = .87; Taris et al., 1999). The reliability for this scale was .92 in this study.

### Job Demands

#### Role Ambiguity and Role Conflict

The two six-item scales developed by Bowling et al. (2017) were utilized. Participants were asked to rate their agreement with the items based on their current job. An example from the role ambiguity scale was “I am not sure what is expected of me at work” and one for role conflict was “I have to deal with competing demands at work.” The reliability for the role ambiguity and role conflict scales was .86 and .94, respectively.

#### Role overload

Originally presented in the Michigan Organizational Assessment Questionnaire (MOAQ; Cammann et al., 1979, 1983), the three-item role overload scale as described by Schaubroeck et al. (1989) was utilized. An example item is “I have too much work to do everything well.” The reliability for this scale was .77.

#### Work-Life Balance

To measure employees’ perceptions of their work-life balance over the last quarter, the four-item measure by Brough et al. (2014) was used. An example item is “Overall, I believe that my work and non-work life are balanced” (α = .83).

### Resources

#### Personal Resilience

The six-item Brief Resilience Scale (Smith et al., 2008) was used to measure resilience. An example item is “I usually come through difficult times with little trouble” (α = .88).

#### Basic Psychological Needs

The need for autonomy (4 items) and relatedness (4 items) subscales from the Basic Psychological Needs at Work scale (Brien et al., 2012) were used in this study. Example items include “My work allows me to make decisions” and “When I’m with the people from work, I feel understood,” respectively. The reliability for these scales was .82 and .93, respectively.

#### Servant Leadership

Originally measured with a twenty-eight-item scale comprising seven dimensions (Liden et al., 2008), servant leadership was measured with the seven-item, short-form version of the original scale, which comprises the highest loading item from each dimension (Liden et al., 2015). An example item is “My leader puts my best interests ahead of his/her own.” The reliability of the long- and short-form are comparable (α = .86–.97; Liden et al., 2008; Liden et al., 2015; and α = .80–.89: Liden et al., 2014; Liden et al., 2015, respectively). The reliability in the present study was .91.

#### Job Opportunities

An adapted version of Turban et al.’s (1992) six-item, perceptions of job attributes subscale (i.e., type of work) which relates to opportunities, was utilized in this study. Participants were asked to report their level of satisfaction (1 = very dissatisfied; 5 = very satisfied) with the type of work in their current position. Adaptations of this measure are noted in several studies (e.g., Ostroff & Clark, 2001; Turban et al., 1998). A typical item is “To use new technology” (α = .91).

#### Pay Satisfaction

A four-item scale from the Pay Satisfaction Questionnaire (PSQ; Heneman & Schwab, 1985) was used to measure pay level satisfaction. Participants were asked to report their level of satisfaction (1 = very dissatisfied; 5 = very satisfied) with their pay. An example item is “My take home pay” (α = .97).

#### Person-Organization Fit

The three-item measure developed by Cable and Judge (1996) was used to assess person-organization fit. Participants assessed their perceived fit by answering each item using a five-point scale ranging from 1 (not at all) to 5 (completely). An example item is “To what degree do you feel your values ‘match’ or fit this organization and the current employees in this organization?” (α = .93).

## Results

### Confirmatory Factor Analysis

Confirmatory factor analyses were conducted using Mplus 7.31 (Muthén & Muthén, 1998–2015) to test the discriminant validity of our constructs. The maximum likelihood estimation method was applied to the covariance matrix to analyze the structure of the data. As shown in Table 1, the 13-factor model displays a good fit to the data (χ^2^_(1691)_ = 3216.29, *p* < .001, CFI = .91, TLI = .91, RMSEA = .05). This model was tested against several alternative models using the chi-square differential test. These more parsimonious models were created by combining two or more factors. The 13-factor model significantly (*p* < .001) improved over all the alternative models. For example, a four-factor model that groups all four demands into one factor and all seven resources into one factor yielded a significantly lower fit (Δχ^2^_(72)_ = 5253.22, *p* < .001) compared to the 13-factor model. Taken together, these results support the discriminant validity of our measures.

**Table 1.** Confirmatory Factor Analysis of Measurement Models: Fit Indices.

	χ^2^	df	CFI	TLI	RMSEA	$\Delta x^{2}$	Δdf
1. Theoretical model with 13 factors	3216.29*	1691	.91	.91	.05	—	—
2. Model with 12 factors							
Grouping relatedness and autonomy	3504.90*	1703	.90	.89	.05	288.61*	12
Grouping turnover intention and emotional exhaustion	3875.74*	1703	.88	.86	.06	659.45*	12
Grouping work-life balance and resilience	4283.59*	1703	.85	.87	.06	1067.30*	12
Grouping work-life balance and autonomy	3845.96*	1703	.88	.85	.06	629.67*	12
Grouping opportunities and pay satisfaction	4224.73*	1703	.87	.85	.06	1008.36*	12
Grouping P-O fit and work-life balance	4055.73*	1703	.87	.86	.06	839.44*	12
3. Model with 11 factors							
Grouping role overload, conflict, ambiguity	3861.98*	1714	.88	.87	.06	645.69*	23
Grouping relatedness, autonomy, opportunities	4200.32*	1714	.86	.85	.06	984.03*	23
4. Model with 10 factors, grouping 4 demands	4511.62*	1724	.84	.83	.06	1295.33*	33
5. Model with 7 factors, grouping 7 resources	7230.61*	1748	.69	.67	.09	4014.32*	57
6. Model with 4 factors, grouping 7 resources and 4 demands	8469.51*	1763	.62	.61	.10	5253.22*	72
7. Model with one factor	10511.66*	1769	.50	.49	.12	7295.37*	78

*Note. N* = 372–373. df = degree of freedom; CFI = comparative fit index; TLI = Tucker-Lewis index; RMSEA = root-mean-square error of approximation.

*p < .001.

### Descriptive Statistics and Correlations

Descriptive statistics and correlations are reported in Table 2. Turnover intention correlated positively with emotional exhaustion (r = .61, *p* < .01), role overload (r = .39, *p* < .01), role ambiguity (r = .44, *p* < .01) and role conflict (r = .44, *p* < .01). Emotional exhaustion correlated positively with role overload (r = .60, *p* < .01), role ambiguity (r = .39, *p* < .01) and role conflict (r = .55, *p* < .01). Turnover intention and emotional exhaustion were negatively related to work-life balance (r = −.46, *p* < .01; and r = −.53, *p* < .01, respectively), resilience (r = −.22, *p* < .01; and r = −.37, *p* < .01, respectively), relatedness (r = −.58, *p* < .01; and r = −.60, *p* < .01, respectively), autonomy (r = −.49, *p* < .01; and r = −.45, *p* < .01, respectively), servant leadership (r = −.57, *p* < .01; and r = −.55, *p* < .01, respectively), job opportunities (r = −.60, *p* < .01; and r = −.51, *p* < .01, respectively), pay satisfaction level (r = −.55, *p* < .01; and r = −.45, *p* < .01, respectively) and person-organization fit (r = −.58, *p* < .01; and r = −.52, *p* < .01, respectively).

**Table 2.** Descriptive Statistics and Correlations for the Study Variables.

Variable	1	2	3	4	5	6	7	8	9	10	11	12	13	*M*	*SD*
Work outcomes															
1. Turnover intention	(.94)													2.32	1.37
2. Emotional exhaustion	.61*	(.92)												2.64	1.13
Job demands															
3. Role overload	.39*	.60*	(.77)											2.37	0.98
4. Role ambiguity	.44*	.39*	.38*	(.86)										1.88	0.79
5. Role conflict	.44*	.55*	.60*	.46*	(.94)									2.63	0.94
6. Work-life balance	–.46*	–.53*	–.53*	–.29*	–.46*	(.83)								3.70	1.02
Resources															
7. Resilience	–.22*	–.37*	–.26*	–.20*	–.19*	.24*	(.88)							3.51	0.93
8. Relatedness	–.58*	–.60*	–.35*	–.47*	–.47*	.40*	.29*	(.93)						3.62	1.06
9. Autonomy	–.49*	–.45*	–.32*	–.50*	–.36*	.41*	.34*	.62*	(.82)					4.19	0.70
10. Servant leadership	–.57*	–.55*	–.36*	–.41*	–44*	.44*	.26*	.67*	.50*	(.91)				3.29	1.01
11. Job opportunities	–.60*	–.51*	–.29*	–.44*	–.29*	.42*	.25*	.59*	.52*	.67*	(.91)			3.52	0.94
12. Pay level satisfaction	–.55*	–.45*	–.35*	–.32*	–.33*	–.35*	.23*	.47*	.30*	.57*	.60*	(.97)		3.07	1.26
13. Person-organization fit	–.58*	–.52*	–.29*	–.36*	–.37*	.43*	.24*	.66*	.52*	.68*	.65*	.51*	(.93)	3.46	1.10

*Note.* On the diagonal and in parentheses are the reliability coefficients (Cronbach’s alpha).

*p < .01.

### Multiple Linear Regressions

To investigate the relations between job demands and resources and turnover intention and emotional exhaustion, two linear regression analyses were conducted. Table 3 presents the linear regression results wherein the standardized beta coefficients are included. First, the overall model with turnover intention as the dependent variable was significant (F (11, 352) = 38.103, *p* < .001, R^2^ = .54). Of the four demands, only work-life balance was significantly related to turnover intention (β = −.090, *p* < .05), while role overload (β = .026, ns), role ambiguity (β = .067, ns) and role conflict (β = .076, ns) were non-significant. The personal resource of resilience was not significantly related to turnover intention (β = .031, ns). In terms of job resources, relatedness (β = −.134, *p* < .05), job opportunities (β = −.189, *p* < .001), pay level satisfaction (β = −.197, *p* < .001) and person-organization fit (β = −.146, *p* < .01) were significantly related to turnover intention. Autonomy (β = −.068, ns) and servant leadership (β = −.031, ns) did not achieve statistical significance. These results provide (partial) support to Hypotheses 4a (work-life balance), 7a (relatedness), 10a (job opportunities), 11a (pay level satisfaction) and 12a (person-organization fit), while Hypotheses 1a (role overload), 2a (role ambiguity), 3a (role conflict), 6a (resilience), 8a (autonomy) and 9a (servant leadership) are rejected.

**Table 3.** Linear Regression of Turnover Intention and Emotional Exhaustion on Job Demands and Resources.

Predictor	Turnover intention	Emotional exhaustion
Job demands		
Role overload	.026	.295***
Role ambiguity	.067	–.047
Role conflict	.076	.144**
Work-life balance	–.090*	–.132**
Resources		
Resilience	.031	–.135***
Relatedness	–.134*	–.243***
Autonomy	–.068	–.015
Servant leadership	–.031	–.012
Job opportunities	–.189***	–.103
Pay level satisfaction	–.197***	–.042
Person-organization fit	–.146**	–.054
R^2^ (adjusted R^2^)	.54 (.53)	.59 (.58)
F	38.103***	46.704***
df	11, 352	11, 352

*Note. N* = 364. Regression weights are reported as standardized coefficient betas.

*p < .05; **p < .01; ***p < .001.

The regression model for emotional exhaustion was significant (F (11, 352) = 46.704, *p* < .001, R^2^ = .59). When considering the demands, role overload (β = .295, *p* < .001) and role conflict (β = .144, *p* < .01) were positively related to emotional exhaustion while work-life balance (β = −.132, *p* < .01) was negatively related to emotional exhaustion whereas role ambiguity (β = −.047, ns) was non-significant. When considering the resources, resilience (β = −.135, *p* < .001) was negatively associated with emotional exhaustion. In terms of job resources, relatedness (β = −.243, *p* < .001) was negatively related to emotional exhaustion while autonomy (β = −.015, ns), servant leadership (β = −.012, ns), job opportunities (β = −.103, ns), pay level satisfaction (β = −.042, ns) and person-organization fit (β = −.054, ns) did not achieve statistical significance. These results provide (partial) support to Hypotheses 1b (role overload), 3b (role conflict), 4b (work-life balance), 6b (resilience), 8b (relatedness), while Hypotheses 2b (role ambiguity), 8b (autonomy), 9b (servant leadership), 10b (job opportunities), 11b (pay level satisfaction) and 12b (person-organization fit) are rejected.

### Relative Weights Analyses

The results of the univariate relative weights analysis are presented in Table 4. First, with turnover intention as the dependent variable, the resource variables of job opportunities (7.7%) and pay level satisfaction (8.3%) account for approximately the same amount of variance, followed by person-organization fit (6.6%) and relatedness (5.9%). Together, these job resources account for over half (28.5%) of the explained (i.e., non-error) variance of turnover intention. On the other hand, the job demand of work-life balance (4.2%) accounted for a modest amount of the explained variance in turnover intention. In sum, the job resources accounted for a much greater amount of variance explained in turnover intention than job demands.

**Table 4.** Relative Weights Analysis Results for Relative Importance of Predictors.

	Turnover intention				Emotional exhaustion			
			95% Confidence interval				95% Confidence interval	
Variable	Raw relative weight	Rescaled weight	Lower bound	Upper bound	Raw relative weight	Rescaled weight	Lower bound	Upper bound
Job demands								
Role overload	0.026	4.95	0.001	0.052	**0.122**	20.39	0.083	0.169
Role ambiguity	0.036	6.90	0.015	0.067	0.020	3.37	0.009	0.041
Role conflict	0.035	6.70	0.016	0.062	**0.075**	12.54	0.048	0.110
Work-life balance	**0.042**	7.92	0.019	0.074	**0.065**	10.75	0.035	0.101
Resources								
Resilience	0.006	1.14	−0.002	0.021	**0.043**	7.21	0.018	0.078
Relatedness	**0.059**	11.28	0.036	0.088	**0.079**	13.21	0.051	0.115
Autonomy	0.043	8.26	0.022	0.073	0.026	4.40	0.013	0.045
Servant leadership	0.051	9.65	0.029	0.077	0.045	7.46	0.027	0.068
Job opportunities	**0.077**	14.70	0.048	0.110	0.046	7.66	0.025	0.074
Pay level satisfaction	**0.083**	15.90	0.050	0.123	0.034	5.60	0.017	0.057
Person-organization fit	**0.066**	12.60	0.038	0.099	0.045	7.42	0.025	0.070

*Note. N* = 364. Turnover Intention (model R^2^ = 0.52); Emotional exhaustion (model R^2^ = .60). Raw Relative Weights in boldface are significant results from Table 3.

In contrast, job demands accounted for a larger portion of explained variance in emotional exhaustion than the resources. Specifically, role overload (12.2%) accounted for the most while role conflict (7.5%) and work-life balance (6.5%) accounted for roughly the same amount of explained variance. Together, these job demands accounted for nearly half (26.2%) of the explained variance in emotional exhaustion. Interestingly, the personal resource of resilience (4.3%) and the job resource of relatedness (7.9%) accounted for a notable amount of explained variance. In sum, job demands accounted for a greater amount of variance explained in emotional exhaustion, the results supporting Hypothesis 5. However, the contribution of resources (i.e., resilience and relatedness) was sizeable as well.

## Discussion

### Theoretical Implications

The present study is among the first to take a demands-resources perspective on both well-being and turnover intention. Our findings indicate that the previously described dual processes may have a degree of cross-over (Bakker & Demerouti, 2017) where both well-being and turnover intention were related to some demands and resources. Partially consistent with Örtqvist and Wincent (2006), two of three role stressors, role overload and role conflict, were positively related to emotional exhaustion. Suggesting that as an employee’s workload increases and conflicting requests are received, their well-being may suffer. An employee’s perception of a good balance between work and personal life was related to lower turnover intention and emotional exhaustion. These findings corroborate work by Brough et al. (2014) and Zhang et al. (2012) suggesting that workplaces that promote work-life balance might benefit from a lower turnover rate and experience fewer consequences related to exhausted employees (e.g., Keeney et al., 2013; Reichl et al., 2014).

As suggested in the literature, job demands may not be directly related to intention to quit (Hu et al., 2011) but rather lead to feelings of exhaustion. The present study noted that only work-life balance, a job demand, has a statistically significant relationship with turnover intention. In contrast, three demands (i.e., role overload, role conflict, and work-life balance) were found to have significant relationships with emotional exhaustion. Thus, employees feeling exhausted may experience a higher intention to quit (e.g., Hu et al., 2011). Resources and job demands appear to have varying strength of influence which may explain why some hypotheses were not supported in the present study and recent empirical work (Upadyaya & Salmela-Aro, 2020). For example, role overload may be a more substantial job demand than role ambiguity which may explain why the latter was not found to be related to emotional exhaustion.

A significant finding of the study is that the four statistically significant job resources (i.e., relatedness, job opportunities, pay level satisfaction and person-organization fit) accounted for over half the explained variance in turnover intention (28.5%). The three statistically significant job demands (i.e., role overload, role conflict and work-life balance) were responsible for almost half of the explained variance in emotional exhaustion (26.2%). The relative importance of the job resources contributing to turnover intention suggests that employees may attribute similar importance to various domains of their job and workplace. For example, an employee wants to easily relate to their coworkers, their values to match with those of the organization, receive satisfactory pay, and be given opportunities to learn and develop on the job. The relative importance of job demands contributing to emotional exhaustion suggests that a workload unfeasible to complete has the most considerable impact on an employee’s well-being. Role conflict and work-life balance are of similar importance.

Finally, personal resilience and having relatable coworkers as job resources negatively contribute to employee ill-being. The findings suggest that effectively managing the stress induced by, for example, a heavy workload, and feeling close with one’s coworkers may reduce the perception of feeling burned out (e.g., Van den Broeck et al., 2008).

### Managerial Implications

Practically speaking, employees with not only challenging (but reasonable) work in a resource-balanced organizational environment appear to perform best in their job (Bakker & Demerouti, 2014). This study suggests that resources bear more heavily as contributors to reduced turnover intention, with demands being more strongly related to employee well-being. Managers would be wise to account for different aspects of the job and work environment having different outcomes. First, a resource-rich organizational environment could potentially reduce employee turnover intention and consequently the rate of voluntary turnover. On the other hand, a demand-heavy organizational environment may lead to employee ill-being, and consequently, employee turnover intention (Richer et al., 2002).

Having too much work and not enough time to complete, known as role overload, was found to be the factor most strongly related to reduced employee well-being. By simply monitoring subordinates’ workloads and deadlines, managers could alleviate the possibility of an employee’s work-related decline in well-being. Moreover, given that both personal resilience and degree of relatedness to coworkers were found to be negatively associated with emotional exhaustion, managers could bolster the personal resilience of employees and team-bonding activities to alleviate the possibility of employees feeling burned out.

### Limitations and Future Research

As with most empirical research in management, the present study utilized self-report measures and data collected at a single time point. According to Podsakoff and colleagues (2003), both of which may be affected by common method variance. Similarly, Baumgartner and Weijters (2012) suggested that method variance bias differs in its impact depending on the source. Both common (i.e., shared method) method variance and uncommon (i.e., unshared) method variance can affect the relationships between different study variables (Spector et al., 2019). The former tends to inflate, and the latter tends to attenuate the magnitude of the relationship between various study variables (Spector et al., 2019; Williams & Brown, 1994).

Using online panel data, where participants are anonymous, it could be suggested that participants may be inclined to answer randomly or untruthfully. Additionally, another limitation with our study is that the participants lived and worked in the U.S., suggesting that the generalizability of the findings needs to be ascertained using further samples. As such, we suggest that the study should be replicated in various regions around the world with a diverse (e.g., tenure, education, and industry) participant pool. Moreover, as the present study utilized a single time point, future research should gather longitudinal data to elucidate the effects of demands and resources over time.

## Conclusion

In conclusion, supporting employees can be challenging, given the multitude of factors at play. For example, the present study evaluated 11 unique variables covering various factors related to the typical work environment. In the scope of the JD-R model (Demerouti et al., 2001), these represent a fraction of the possible demands, resources and outcomes that could influence one another. As such, this study offers an empirical approach to discerning the influential factors relating to employee turnover intention and their well-being. Specifically, the present study contributes to the vast JD-R model-centred literature by being among the first to elucidate the relative importance of various demands and resources with two distinct work-related outcomes: turnover intention and emotional exhaustion. Including a personal resource, job-related resources, and job demands advances JD-R theory to include the potential impact of employee characteristics. Rather than viewing various demands and resources as equal, this study suggests a unique impact of each demand and resource on employee turnover intention and well-being. The inclusion of turnover intention, which could be viewed as a more consequential form of disengagement behavior, allows for further development of the JD-R model.
